# Supplementary material for: Elucidating the Subcellular Localization of GLRaV-3 Proteins Encoded by the Unique Gene Block in N. benthamiana Suggests Implications on Plant Host Suppression
Source: Biomolecules. 2024 Aug 9;14(8):977. doi: 10.3390/biom14080977 (PMC11352578; doi:10.3390/biom14080977)
Supplement: Supplementary file 1 [file biomolecules-14-00977-s001.zip › biomolecules-3111073-supplementary.pdf]

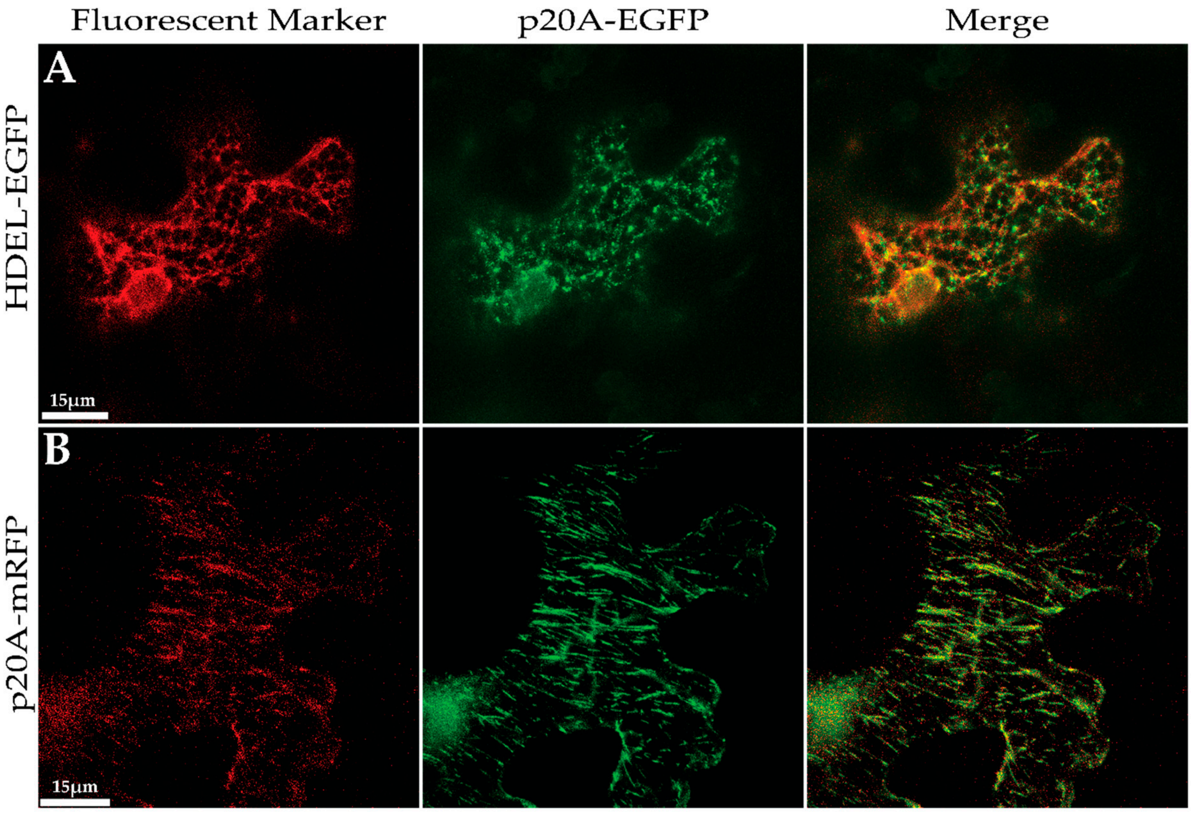

Supplemental Figure S1. Infiltration culture concentration dependent behaviour of p20A-EGFP. A. N. benthamiana leaf tissue agro-infiltrated with HDEL-mRFP and p20A-EGFP. Green and red channels are overlayed in rightmost panel. B. N. benthamiana leaf tissue agro-infiltrated with p20A-mRFP (at an OD600 of 0.237) and p20A-EGFP (at an OD600 of 0.031). Green and red channels are overlayed in rightmost panel.

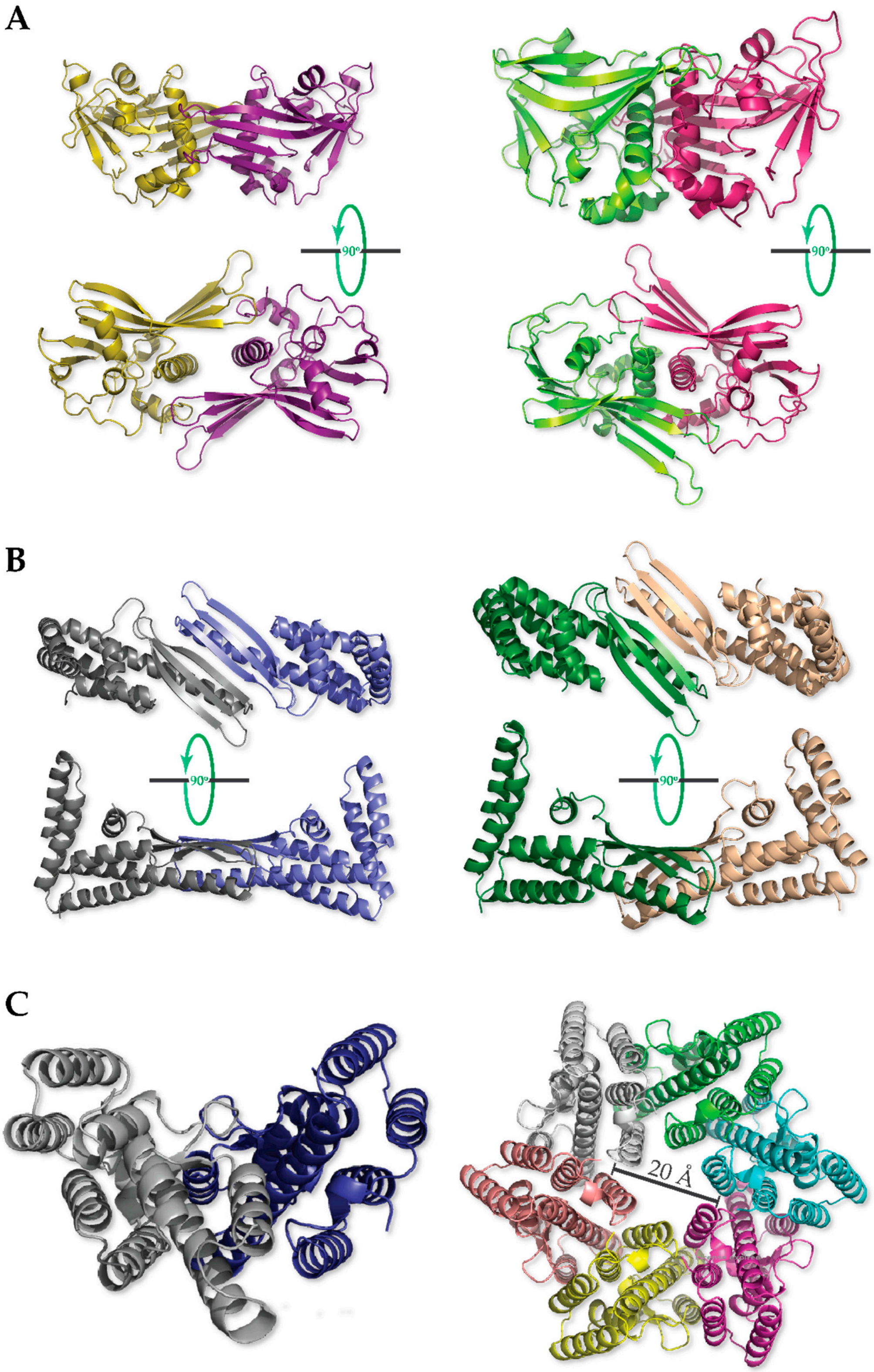

Supplemental Figure S2. Homomer conformations of A. p21, B. p20A and C. p20B as predicted by Galaxy Homomer. Two oligomeric states with highest docking scores shown for each viral protein.

Supplemental Table S1. Fusion proteins and their relative optical density (OD_{600}) used in agrobacterium mediated genetic transformation.

Infiltrated Protein	Optical Density (OD_{600})	Fluorescent Protein
p21-EGFP	0.233	Enhanced GFP (Clontech) [100]
mRFP	0.240	Monomeric RFP [101]
p20A-EGFP	0.211	Enhanced GFP (Clontech) [100]
p20A-mRFP	0.237	Monomeric RFP Monomeric RFP [101]
p20B-EGFP	0.05	Enhanced GFP (Clontech) [100]
p20B-EGFP-GUS	0.1	Enhanced GFP (Clontech) [100]
p20B-ΔNLS2-EGFP-GUS	0.1	Enhanced GFP (Clontech) [100]
p20B-ΔNLSε-EGFP-GUS	0.1	Enhanced GFP (Clontech) [100]
NLS-EGFP-GUS	0.1	Enhanced GFP (Clontech) [100]
HDEL-mRFP	0.162	Monomeric RFP [101]
CD3-987	0.169	sGFP [102]
mTalin-GFP	0.165	mGFP4 [103]
MBD-GFP	0.2	sGFP [102]
pCP:mRFP	0.2	Monomeric RFP Monomeric RFP [101,104]

Supplemental Table S2. Homomer Prediction of GLRaV-3 p21, p20A and p20B by Galaxy Homomer.

Model Number	Number of Subunits	Interface Area	Docking Score
1 p21	2-mer	975.1	910.664
2 p21	2-mer	1635.1	793.878
3 p21	2-mer	1090.4	783.688
4 p21	2-mer	837.3	765.738
5 p21	4-mer	2700.3	640.999
1 p20A	2-mer	851.9	1117.024
2 p20A	2-mer	844.8	891.338
3 p20A	2-mer	1158.3	798.803
4 p20A	6-mer	5872.9	674.601
5 p20A	10-mer	5956.8	648.474
1 p20B	2-mer	1559.0	1022.224
2 p20B	6-mer	8303.6	731.318
3 p20B	3-mer	2738.6	697.871
4 p20B	3-mer	3062.7	635.702
5 p20B	3-mer	1896.9	627.577
